# Antimicrobial and Antibiofilm Activity and Machine Learning Classification Analysis of Essential Oils from Different Mediterranean Plants against *Pseudomonas aeruginosa*

**DOI:** 10.3390/molecules23020482

**Published:** 2018-02-23

**Authors:** Marco Artini, Alexandros Patsilinakos, Rosanna Papa, Mijat Božović, Manuela Sabatino, Stefania Garzoli, Gianluca Vrenna, Marco Tilotta, Federico Pepi, Rino Ragno, Laura Selan

**Affiliations:** 1 Department of Public Health and Infectious Diseases, Sapienza University, P.le Aldo Moro 5, 00185 Rome, Italy; marco.artini@uniroma1.it (M.A.); rosanna.papa@uniroma1.it (R.P.); gianluca.vrenna@uniroma1.it (G.V.); marco.tilotta@uniroma1.it (M.T.); laura.selan@uniroma1.it (L.S.); 2 Department of Drug Chemistry and Technology, Sapienza University, P.le Aldo Moro 5, 00185 Rome, Italy; alexandros.patsilinakos@uniroma1.it (A.P.); mijatboz@gmail.com (M.B.); manuela.sabatino@uniroma1.it (M.S.); stefania.garzoli@uniroma1.it (S.G.); federico.pepi@uniroma1.it (F.P.); 3 Rome Center for Molecular Design, Department of Drug Chemistry and Technology, Sapienza University, P.le Aldo Moro 5, 00185 Rome, Italy; 4 Alchemical Dynamics s.r.l., 00125 Rome, Italy; 5 Faculty of Natural Sciences and Mathematics, University of Montenegro, Džordža Vašingtona bb, 81000 Podgorica, Montenegro

**Keywords:** biofilm, *Pseudomonas aeruginosa*, essential oil, machine learning, antibacterial

## Abstract

*Pseudomonas aeruginosa* is a ubiquitous organism and opportunistic pathogen that can cause persistent infections due to its peculiar antibiotic resistance mechanisms and to its ability to adhere and form biofilm. The interest in the development of new approaches for the prevention and treatment of biofilm formation has recently increased. The aim of this study was to seek new non-biocidal agents able to inhibit biofilm formation, in order to counteract virulence rather than bacterial growth and avoid the selection of escape mutants. Herein, different essential oils extracted from Mediterranean plants were analyzed for their activity against *P. aeruginosa*. Results show that they were able to destabilize biofilm at very low concentration without impairing bacterial viability. Since the action is not related to a bacteriostatic/bactericidal activity on *P. aeruginosa*, the biofilm change of growth in presence of the essential oils was possibly due to a modulation of the phenotype. To this aim, application of machine learning algorithms led to the development of quantitative activity–composition relationships classification models that allowed to direct point out those essential oil chemical components more involved in the inhibition of biofilm production. The action of selected essential oils on sessile phenotype make them particularly interesting for possible applications such as prevention of bacterial contamination in the community and in healthcare environments in order to prevent human infections. We assayed 89 samples of different essential oils as *P. aeruginosa* anti-biofilm. Many samples inhibited *P. aeruginosa* biofilm at concentrations as low as 48.8 µg/mL. Classification of the models was developed through machine learning algorithms.

## 1. Introduction

The great ability of bacteria to colonize new environments is undoubtedly related to biofilm formation. Biofilm lifestyle is associated with a high tolerance to exogenous stress: consequently, the treatment of biofilms with antibiotics or other biocides is usually ineffective at eradicating them. Biofilm formation is inevitably a major problem in many fields, ranging from food industry to medicine. In medical settings, biofilms are the cause of persistent infections implicated in 80% or more of all microbial cases, releasing harmful toxins and even obstructing indwelling catheters [1]. Bacteria of clinical relevance—such as *Pseudomonas aeruginosa*, *Staphylococcus aureus*, and *Acinetobacter baumannii* among others—proliferate on medical devices and form biofilms which provide them with up to 1000 times more effective resistance and tolerance to antibiotics in comparison with their planktonic forms [2].

*P. aeruginosa* is a common Gram-negative bacillus, able to adapt and survive in unfavorable environmental conditions including minimal nutritional sources. It can cause disease in plants and animals, as well as humans. *P. aeruginosa* is a multidrug resistant pathogen recognized for ubiquity, intrinsically advanced antibiotic resistance mechanisms, and association with serious illnesses—especially hospital-acquired infections such as ventilator-associated pneumonia (VAP) [3] and various sepsis syndromes [4]. Serious infections caused by *P. aeruginosa* often occur during existing diseases or conditions—most notably cystic fibrosis and traumatic burns. In spite of the progress of antimicrobial therapies, infections by *P. aeruginosa* can still cause a mortality percentage range between 18% and 61% of cases [5,6]. The great impact of *P. aeruginosa* infection is mainly due to its capability to form biofilm [7].

Once firmly established, the biofilm can be very difficult to eradicate as the bacteria are embedded in a self-produced polymeric substance, providing poor susceptibility to conventional antimicrobial agents [8] and host defense cells of immunologic system and resulting in chronic infections [9].

Considering these assumptions, the interest in the development of new approaches for the prevention of bacterial adhesion and biofilm formation has increased. The development of anti-biofilm strategies is therefore of major interest and currently constitutes an important field of investigation in which non-biocidal molecules are highly valuable to avoid the rapid appearance of escape mutants [10].

Therefore, the rationale of this study was to search for new antimicrobials which have the power to inhibit virulence instead of bacterial growth; such a choice may impose a weaker selective pressure for the development of antibiotic resistance to current antibiotics.

Compounds of natural origin still provide a high number of interesting structures, even in this era of combinatorial chemistry.

Essential oils (EOs) represent a group of antimicrobial agents which are complex mixtures of volatile secondary metabolites [11,12].

EOs show antimicrobial and antifungal properties and are also largely used in various cultures for medical and health purposes. The wide use of EOs apply to aromatherapy, household cleaning products, personal beauty care, and natural medical treatments.

Furthermore, EOs may synergically enhance the antimicrobial potencies of some drugs [13,14].

Recently, several EOs and phytochemicals have been reported to inhibit biofilm formation by bacteria and fungi [15,16], and their effects on *P. aeruginosa* have been studied [17,18]. Taking into consideration the same plant variety, EO composition can differ according to geographical region, seasonality, and extraction methodology [19,20,21].

This study reports chemical composition, antibacterial and anti-biofilm activity against *P. aeruginosa* of 89 different EOs obtained from 3 different plants harvested in different seasons and conditions: *Calamintha nepeta* (L.) Savi subsp. *glandulosa* (Req.) Ball (CG) [19], *Foeniculum vulgare* Miller (FV) [21], and *Ridolfia segetum* Moris (RS). Furthermore, quantitative activity–composition relationships (QCAR) were developed through machine learning classification approaches with objective of discovery the chemical components mainly responsible for the anti-biofilm activity.

## 2. Material and Methods

### 2.1. Plant Materials

As previously described, fresh aerial parts of CG [19], FV [21], and RS were collected in a wild area about 15 km from Tarquinia city (Province of Viterbo, Italy), in the archaeological zone near the Etruscan temple Ara della Regina (42°15′31.8″ N, 11°48′08.7″ E). The material was collected in summer and early autumn periods of the year 2015 and monitored for four (CG) and three (FV) months, from July to October, thus covering pre-, during-, and post-flowering periods. Regarding RS, this is a summer annual plant, completing its life cycle within June/July. Hence, this species has been monitored just in terms of different extraction times, not periods of harvesting. CG oils were obtained directly from fresh plant material, while FV and RS were air-dried in a shady place for 20 days. Voucher specimens have been deposited in the Department of Drug Chemistry and Technology at Sapienza University of Rome, Italy. Taxonomic identification of the chosen species was conducted according to the official European flora and the National Italian one.

### 2.2. EO Extraction

EOs have been isolated, as previously reported, by direct steam distillation using a 62 L steel distillator apparatus (Albrigi Luigi E0131, Verona, Italy) [19,20,22]. Briefly, plant materials (about 1.5 kg) were subjected to fractioned steam distillation [20], collecting EOs at six interval times of 1, 2, 3, 6, 12, and 24 h, in case of RS a seventh fraction was collected after 30 h.

At each fraction, the oil/water double phase was extracted three times with 20 mL of diethyl ether. The organic layers were dried over anhydrous sodium sulfate (Na_2_SO_4_), filtered, and deprived of the solvent in vacuo to furnish the final EOs, which were stored in freezer in tightly closed dark vials until further analysis.

In addition, to simulate parallel continuous EO extraction for 2, 3, 6, 12, and 24 h (and 30 h), mixtures were prepared by adding different amounts of diethyl ether to each oil fraction, up to 10 mL in total (e.g., 7 mL of diethyl ether to 3 mL of the oil). Combining 1 mL of each ether–oil solution and then letting ether to evaporate, the desired oily mixes were obtained.

### 2.3. GC–MS Analysis

The gas chromatographic/mass spectrometric (GC/MS) EOs analyses were carried out with a GC-MS and GC-FID similarly as previously described [19,22].

### 2.4. Bacterial Strains and Culture Conditions

*P. aeruginosa* PaO1 was grown in Brain Heart Infusion broth (BHI, Oxoid, Basingstoke, UK). Planktonic cultures were grown in flasks under vigorous agitation (180 rpm) at 37 °C while biofilm formation was assessed in static condition at 37 °C in a 96-well plates for 18 h.

### 2.5. Determination of Minimal Inhibitory Concentration (MIC)

MIC was performed according to the guidelines of Clinical Laboratory Standards Institute (CLSI). Each EO was added directly from mother stock and solutions were prepared by two-fold serial dilutions. Mother stock solutions were obtained by solubilizing each EO in DMSO at a final concentration of 1 g/mL. A total of 10 concentrations were used within the 25–0.045 mg/mL range. Experiments were performed in quadruplicate. The MIC was determined as the lowest concentration at which the observable bacterial growth was inhibited. No inhibition of the bacterial growth was highlighted at tested concentrations.

### 2.6. Static Biofilm Assay

Biofilm formation of *P. aeruginosa* PaO1 was evaluated in the presence of each EO. Quantification of in vitro biofilm production was based on previously reported methodology [10]. Briefly, the wells of a sterile 96-well flat-bottomed polystyrene plate were filled with 100 µL of the appropriate medium. A measure of 1/100 dilution of overnight bacterial cultures was added into each well (about 0.5 OD 600 nm). As a control, the first row contained bacteria grown in 100 μL of BHI (untreated bacteria). Furthermore, BHI broth was added to remaining wells starting from the third row. In the second row, we added BHI supplemented with each EO at a concentration of 25 mg/mL. Starting from this lane, samples were serially diluted (1:2 dilutions). The plates were incubated aerobically for 18 h at 37 °C.

Biofilm formation was measured using crystal violet staining. After treatment, planktonic cells were gently removed; each well was washed three times with double-distilled water and patted dry with a piece of paper towel in an inverted position. To quantify biofilm formation, each well was stained with 0.1% crystal violet and incubated for 15 min at room temperature, rinsed twice with double-distilled water, and thoroughly dried. The dye bound to adherent cells was solubilized with 20% (*v*/*v*) glacial acetic acid and 80% (*v*/*v*) ethanol. After 30 min of incubation at room temperature, OD590 was measured to quantify the total biomass of biofilm formed in each well. Each data point is composed of four independent experiments, each performed at least in triplicate.

### 2.7. Statistical Analysis of Biological Evaluation

Data reported were statistically validated using Student’s *t*-test comparing mean absorbance of treated and untreated samples. The significance of differences between mean absorbance values was calculated using a two-tailed Student’s *t*-test. A *p* value of <0.05 was considered significant.

### 2.8. Machine Learning Binary Classification

#### 2.8.1. General Methods

Binary classification models development and validation were carried out by an in-house python script based on the scikit-learn machine learning library [23]. First, the data were imported and pre-processed to obtain the independent data matrix consisting of 89 rows (essential oil samples) and 54 columns (chemical components). Two dependent target vectors containing 89 biofilm formation percentage observations at 48 μg/mL and 3.125 mg/mL were defined.

Principal component analysis (PCA) [24] was used to check for linear data separability, while gradient boosting (GB) [25] for non-linear classification. Cross-validation was used to search for the optimal inhibition percentage cut-off value in order to define active and inactive samples. The optimal cut-off values were used to obtain the final classification model. Hyper-parameter optimization was finally achieved through a systematic grid search of number of stages to perform (number of trees), maximum depth of individual tree which limits the number of nodes in the tree (max depth), minimum number of samples required to be at a leaf node (min sample leaf), and the number of features to consider (Supplementary Materials Table S1).

The final classification model was numerically and graphically evaluated by accuracy (ACC), Matthews correlation coefficient (MCC), receiver operating characteristic (ROC), and precision–recall (PR) curves (Supplementary Materials Figure S2). Finally, the importance of EOs chemical components was evaluated individually by means of the “feature importance” and “partial dependence” plots [25]. Partial dependence plots may be viewed as a graphical representation of linear regression model coefficients that extends to arbitrary model types, addressing a significant component of the model.

#### 2.8.2. Validation

Validation of the classification model was carried out by leave-one-out cross-validation and taking into account the accuracy (ACC), the precision or positive predictive value (PPV), the recall or sensitivity or true positive rate (TPR), specificity or true negative rate (TNR), receiver operating characteristic (ROC) curve, and the Matthews correlation coefficient (MCC) [26].

#### 2.8.3. Accuracy (ACC)

ACC (Equation (1)) is the proportion of true positives (TP) and true negatives (TN) among the total cases examined (P + N). That is, the ratio of correct classifications to the total number of correct or incorrect classifications.

(1) $$\mathrm{ACC}=\;\frac{\mathrm{TP}\;+\;\mathrm{TN}}{P+\;N}$$

#### 2.8.4. Precision, Positive Predictive Values (PPV)

PPV (Equation (2)) describes the ability of the classifier not to label as positive a sample that is negative. That is, given a positive prediction, how likely is the classifier to be correct. The best value is 1 and the worst value is 0.

(2) $$\mathrm{PPV}\;=\;\frac{\mathrm{TP}}{\mathrm{TP}\;+\;\mathrm{FP}}$$

#### 2.8.5. Recall, Sensitivity, True Positive Rate (TPR)

TPR (Equation (3)) measures the proportion of positive predicted labels which are correctly identified as such. That is, the ability of the classifier to find all the positives samples. The best value is 1 and the worst value is 0.

(3) $$\mathrm{TPR}\;=\;\frac{\mathrm{TP}}{P}\;=\;\frac{\mathrm{TP}}{\mathrm{TP}\;+\;\mathrm{FN}}$$

#### 2.8.6. TNR

TNR (Equation (4)) measures the proportion of negative predicted labels that are correctly identified as such. That is, is the ability of the classifier to find all the negative samples. The best value is 1 and the worst is 0.

(4) $$\mathrm{TNR}=\;\frac{\mathrm{TN}}{N}=\;\frac{\mathrm{TN}}{\mathrm{TN}\;+\;\mathrm{FP}}$$

#### 2.8.7. Receiver Operating Characteristic (ROC) Curve

The ROC curve [27] shows the ability of a binary classifier model to discriminate between positive and negative classes as its discrimination threshold is varied from high to low values. To obtain the curve, the true positive rate (TPR) is plotted against the false positive rate (FPR) at various thresholds. An area under the curve (AUC) of the ROC curve of 1.0 represents a model that made all predictions correctly. An AUC of 0.5 represents a model that is as good as a random classification.

#### 2.8.8. Matthews Correlation Coefficient (MCC)

MCC (Equation (5)) [26] is a measure of the quality of binary (two-class) classifications. The best value is 1 and the worst value is 0.

(5) $$\mathrm{MCC}\;=\;\frac{\mathrm{TP}\;\times \;\mathrm{TN}\;-\;\mathrm{FP}\;\times \;\mathrm{FN}}{\sqrt{\left(\mathrm{TP}\;+\;\mathrm{FP})(\mathrm{TP}\;+\;\mathrm{FN})(\mathrm{TN}\;+\;\mathrm{FP})(\mathrm{TN}\;+\;\mathrm{FN}\right)}}$$

It takes into account TP, FP, FN, TN, and false positives and negatives and is generally regarded as a balanced measure which can be used even if the classes are of very different sizes. The MCC is in essence a correlation coefficient value between −1 and +1. A coefficient of +1 represents a perfect prediction, 0 an average random prediction, and −1 an inverse prediction.

Only in the binary case does this relate to information about true and false positives and negatives.

## 3. Results

### 3.1. EO Extraction

Fractioned extraction process applied to three different plant species, two of them being also monitored in terms of different harvesting periods, showing great differences in EO yields. Detailed overview of those results has been reported just recently [20]. In the case of CG, usually the largest parts of EOs were extracted in the first 3 or 6 h [19]. Great impact of harvesting period is particularly obvious in the case of FVEOs, and a great increase, of up to five times, in EO amount was noticed in October when the plant was fruiting [21]. Annual RS gave a very unusual yield curve with the first maximum after the first hour of extraction, and the second one between the third and sixth hour of the extraction process. Relative yield percentages of EOs (calculated per weight of fresh/dried plant material) and total yields over time are given in Supplementary Materials Tables S2 and S3.

### 3.2. GC-MS Analysis of EOs

Obtained CGEOs, FVEOs, and RSEOs were analyzed in terms of chemical composition [19,21]. The extraction method applied gave fractions that differ greatly in their chemical composition characterized by 89 samples with a total of 54 chemical constituents differently distributed (Supplemental Materials Tables S4 and S5). For each EO, the main characterizing compounds are usually present in every fraction, variations in their amount are particularly abundant in the first three fractions (up to 3 h of extraction process) with very low percentage, or even absent, in the last three (after 12 h or 24 h). Furthermore, some compounds appear only with the development of the extraction process, being significantly present only in the last fractions. Concerning the harvesting period, EOs chemical profiles were found to be heavily influenced by this factor. Details for CG and FV have been already reported [19,21] (Supplemental Materials Tables S4 and S5), while chemical data for RS are reported here (Supplemental Materials Table S3) but will be detailed and discussed elsewhere.

### 3.3. Qualitative Analysis of EOs Effect on Biofilm Formation of P. aeruginosa

In order to exclude if selected EOs contained molecules affecting bacterial viability, the 89 EOs were analyzed also for antimicrobial activity. In vitro EOs bacteriostatic and bactericidal activities were evaluated on *P. aeruginosa* by broth microdilution methods. An appropriate dilution (10^6^ cfu/mL were used as reported by National Committee for Clinical Laboratory Standards NCCLS, 2004) of bacterial culture of *P. aeruginosa* in exponential phase was used. No antimicrobial activity on *P. aeruginosa* strains was highlighted for all tested EOs (maximal concentration tested 25 mg/mL).

**Figure 1:**
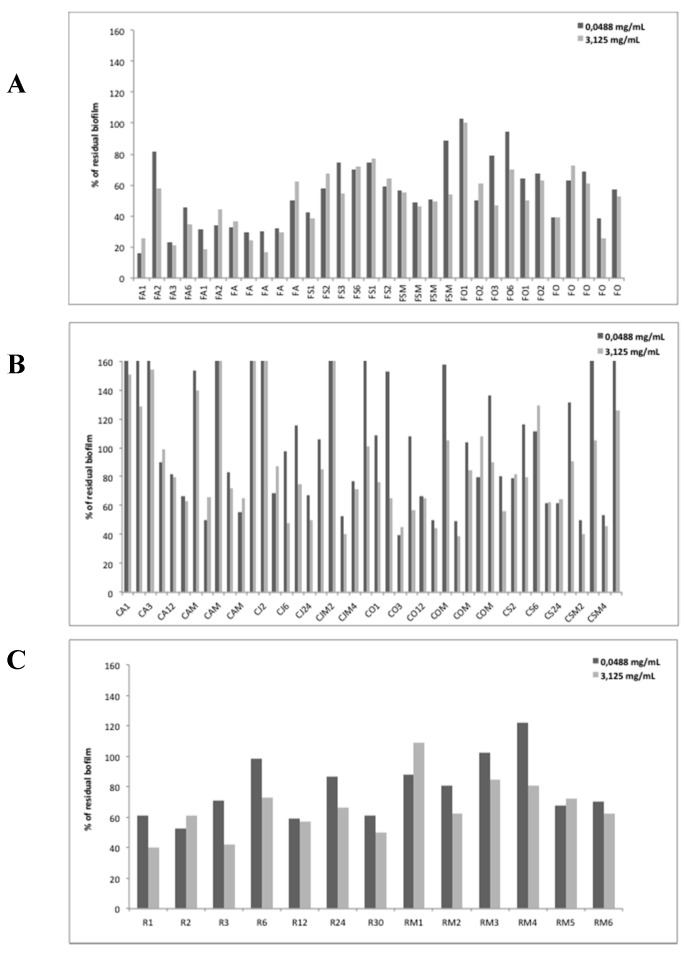
Effect of EOs from *Foeniculum vulgare* Miller (FV) (**A**), *Calamintha nepeta* (L.) Savi subsp. *glandulosa* (Req.) Ball (CG) (**B**), and *Ridolfia segetum* Moris (RS) (**C**) on biofilm formation of *P. aeruginosa* PaO1. Data are reported as percentage of residual biofilm after the treatment in comparison with the untreated one. Each data point is composed of four independent experiments each performed at least in triplicate.

The anti-biofilm effects of EOs from different plants above described were examined on *P. aeruginosa* PAO1. Firstly, we selected some representative EOs samples (two for RS, three for CG and FV) to evaluate the anti-biofilm efficacy at different concentrations starting from 25 mg/mL using scalar dilutions (Table 1). The obtained preliminary data, were analyzed in terms of biofilm reduction and reproducibility that led to the selection of two concentrations as the most representatives (3.125 mg/mL and 0.0488 mg/mL). The first concentration was in the range of milligrams while the second one was in the range of micrograms. The percentages of residual biofilm after treatment at these two concentrations (3.125 mg/mL and 0.0488 mg/mL) for all 89 Eos are reported in Figure 1.

It is worth noticing that each EO had a specific effect on biofilm formation, thus depending on its characteristic and unique composition which was previously quali-quantitatively analyzed chemically. Furthermore, the majority of tested EOs had an inhibitory effect on *P. aeruginosa* PaO1 biofilm formation.

Arbitrarily, three levels of biofilm inhibition were considered to qualitatively cluster the EOs potencies: strong biofilm inhibition in the range 0–40% of residual biofilm, mild inhibition in the range 40–80% and no biofilm inhibition over 80% of residual biofilm, respectively. In some cases, an increase of biofilm formation was highlighted after the treatment. As reported in Figure 1A, almost all EOs samples derived from FV showed to be able in inhibiting biofilm formation of *P. aeruginosa* PaO1, the only exception was the FO1 sample.

Only in such cases a marked effect dose-dependent was observable (i.e., FA2, FSM5, FO3, FO6, and FOM4, where anti-biofilm effect was proportional to the concentration of EO used).

In Figure 1B, the effects of EOs from CG on PaO1 biofilm formation are reported. Differently from FV data, several CGEOs samples showed to increase biofilm production in a way directly proportional to the concentration used. Among all assayed EOs, some of them, such as CO_2_, produced an inhibitory effect on the biofilm at higher concentration and an increase of it at lower concentration. Instead, other EOs are able to strongly inhibit biofilm formation already at very low concentrations (reduction of biofilm higher than 50%).

Regarding the results obtained with extracts derived from RS, all of them inhibited biofilm. In most cases, the reduction is proportional to the concentration of EO used (R1, R3, R6, R24, R30, RM2, RM3, and RM4). Conversely, EOs named R2, R12, RM5, and RM6 did not show an anti-biofilm effect correlated to the concentration used. Only in the case of RM1, there is an opposite relationship between the concentration used and the anti-biofilm effect (Figure 1C).

In Supplementary Materials Table S6 are summarized results on anti-biofilm activity of EO grouped in four different classes corresponding to their capability to impair biofilm formation. This classification was based on results reported in Figure 1 obtained with higher concentration of EOs (3.125 mg/mL). In particular, we considered strong reduction if the residual biofilm was in the range 0–40%, medium reduction if the residual biofilm was in the range 40–80%, and no reduction for residual biofilm higher than 80%. In such cases, an enhancer effect on biofilm formation was evidenced after the treatment with EOs. This latter was observable only for such EOs obtained from CG.

### 3.4. Application of Machine Learning Algorithms

**Figure 2:**
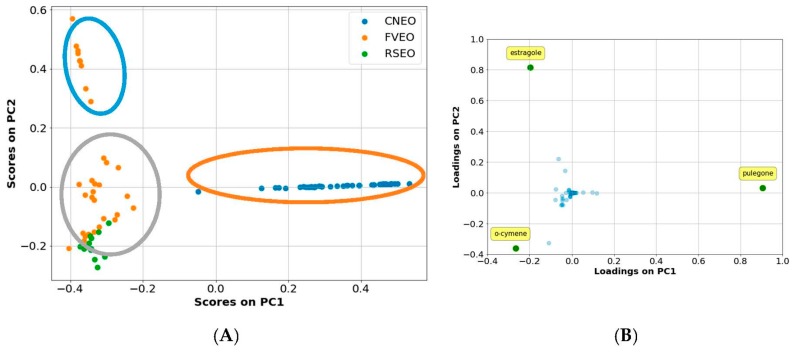
PCA first 2 PCs graphical plots. The core plot (**A**) indicates the presence of at least three clusters (circled in (**A**)). The loading plots (**B**) highlights that estragole, o-cymene, and pulegone could be the most important chemical constituents among all the tested EOs.

Initial application of linear models by the PCA formalism revealed the lack of linear dependence between biofilm production inhibition and chemical composition as no acceptable classification model was obtained using biofilm production percentages observed at either 0.0488 or 3.125 mg/mL. Indeed, graphical inspection of scores plot of the first two principal components (PCs), accounting for more than 75% of data variance, revealed the presence of at least three clusters (Figure 2).

At the same time, from Figure 2, PCA well identified the three plants derived essential oils, although it was not possible to obtain a clear separation between *Ridolfia* and *Foeniculum* EOs. PCA-related loading plots indicated that estragole, o-cymene, and pulegone chemical components were the most important chemical constituents among all the tested EOs. Furthermore, scores plot (Figure 2A) highlighted the lack of any linear classification among the 89 EOs as the three clusters cannot be associated to any level of biofilm production percentage. Linear classification models using algorithms such as logistic regression (LR) and linear support vector machines [28] did no lead to satisfying classifiers (data not shown). Therefore, non-linear algorithms like random forest (RF) [29], non-linear support vector machine (SVM) [25,30] and gradient boosting (GB) were applied.

Among used algorithm (data not shown), GB led to the most robust binary classification model. To this aim, at first the optimal biofilm production percentage for the binary classification was investigated by systematically increasing it from a starting 40 to 80% and monitoring the accuracy by leave-one-out cross-validation. The best GB classification model was obtained at 50% and 46% for the 48.8 µg/mL and 3.125 mg/mL concentrations, respectively (Supplementary Materials Figure S1). Therefore, EOs characterized by more than 50% (or 46%) of biofilm production were classified as inactive, while those with lower values were considered active.

The two models were characterized by satisfactory statistical values (Table 2, Supplementary Materials Figures S2 and S3). In particular a greater robustness was obtained for the classification model defined at 48.8 µg/mL oil concentration as highlighted from ACC, MCV, precision–recall AUC, and ROC AUC higher values (Supplementary Materials Figures S2 and S3).

## 4. Discussion

The aim of this study was to address the potential of selected EOs to prevent and treat biofilm produced by *P. aeruginosa*. This microorganism is widespread in nature and is a frequent foodborne pathogen. Although *P. aeruginosa* is an opportunistic pathogen and rarely cause disease in healthy persons, it is a notorious nosocomial pathogen, posing a high risk to immunosuppressed individuals and other highly vulnerable patients [31]. *P. aeruginosa* can cause pneumonia, catheter-associated and urinary tract infections, and sepsis in wounded patients, sometimes resulting in serious chronic infections and health complications.

The ability of *P. aeruginosa* to form biofilm renders it refractory to the action of antibiotics and disinfectants and also able to survive in unfavorable conditions for a long time.

Based on these considerations, it is clearly evident the importance to have new strategies to impair biofilm formation by *P. aeruginosa*.

### 4.1. Chemical Quantitative Composition–Activity Relationships

Significant to moderate biofilm reducing activity was observed for several CGEO samples against *P. aeruginosa* PaO1 strain. Analyzing data showed in Figure 1, the duration of extraction process seems to influence the activity on this strain, since in every month (except October) last fractions (12 h and 24 h) were found to be more effective. Observed efficacy of these last fractions could be potentially associated with the increase of chrysanthenone.

Some FVEO samples from August harvest demonstrated very high biofilm inhibition of this strain, in some samples even more than 80%. With few exceptions, September samples did not show any significant reduction, while several FVEOs obtained from fruiting material (FOM1 and FOM4) showed significant ability to impair biofilm formation, even in the quite low concentration.

Further data analysis (Figure 2) suggests the potential influence of different chemical profile on oil’s efficacy. Namely, October samples differ from the ones obtained during the pre-fruiting phenological stage, having estragole as the major constituent. The presence of this phenylpropene in these samples may be the main reason for biofilm inhibition. On the other side, the higher susceptibility of *P. aeruginosa* strain to the oils obtained from August-harvesting, may be influenced by some other components characteristic of the EOs obtained in that period that possibly exerts some additive effect in the expression of overall activity (α-phellandrene, β-phellandrene, or thymol).

*P. aeruginosa* PaO1 was sensitive only to certain RSEO fractions (panel C of Figure 1). However, taking into account the chemical analysis of the samples, a positive correlation cannot be established between the content of o-cymene and apiol, the main characterizing compounds, and the inhibition of biofilm formation. Probably some minor components may influence biofilm inhibition.

In such cases, in particular in presence of EOs obtained by CG, an enhancer effect on biofilm formation was observed. This second is an interesting result and support the theory that plants produce molecules that regulate biofilm formation in different environments: an interesting example of inter-kingdom regulation. The regulatory pathways of sessile phenotype could be related to competition dynamics of habitats. The identification of the molecules responsible for these mechanisms could be interesting and also open new perspectives for the control of bacterial biofilm formation. It is worth to note that ‘row’ EOs that we used represent a complex pool of chemical cues that could be characterized by different capabilities to either impair or promote biofilm formation.

### 4.2. Gradient Boosting Binary Classification Model

**Figure 3:**
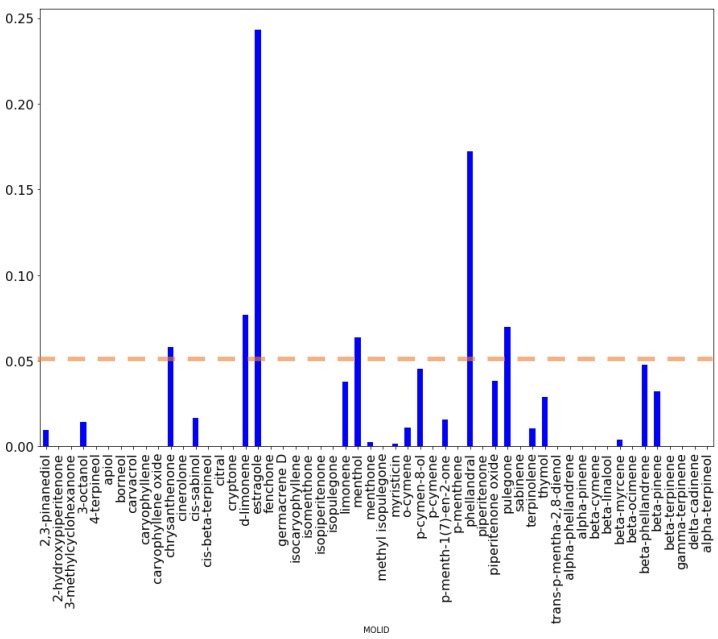
Feature importance plot obtained for the GB classification models at 48.8 µg/mL.

The most robust classification model defined at 48.8 µg/mL oil concentration was analyzed by means of feature importance and partial dependence plots [25] (Figure 3 and Supplementary Materials Figure S4). Feature importance plot highlights the absolute importance of each chemical constituent while partial dependence plots built for the most important components gives direct univariate relationships with the biofilm inhibitory activity, giving direct information on positive or negative effects.

From Figure 3, considering a threshold of 5%, six compounds—namely estragole, methol and phellandral, d-limonene, pulegone, and chrysanthenone—can be considered as those most influencing biofilm production, being estragole and phellandral the most significant. To ascertain whether components correlate positively or negatively with biofilm inhibition, the partial dependence plot were investigated for all compounds (Supplementary Materials Figure S4). The partial dependent plots for the above six compounds (Supplementary Materials Figure S5) directly indicate estragole and phellandral as those most important for biofilm inhibition. Whereas d-limonene, pulegone, and chrysanthenone were found to be inversely associated to biofilm inhibition and likely responsible for biofilm production enhancement observed. A different scenario can be interpreted for menthol, at a low percentage it correlates with a negative effect on biofilm inhibition that disappears as it is increases above 16%.

## 5. Conclusions

In this study, it was demonstrated that biofilm growth of *P. aeruginosa* PAO1 is influenced by the presence of EOs extracted from three different Mediterranean plants harvested in different seasons.

Our results suggest that the kind of biofilm modulation depends on EO chemical composition although the fractions were obtained from the same plant. Remarkably, an important influence on the modulation of biofilm production is related to the harvesting period. Furthermore, in some cases, the same EOs seem to exert opposite influences (stimulation or inhibition of biofilm growth) depending on sample dilution. This is clearly related to the concentration of specific chemical compound as highlighted by the classification models. Since the action is not related to a bacteriostatic/bactericidal activity on *P. aeruginosa*, the biofilm change of growth in presence of the EOs is possibly due to a modulation of the phenotype that switches from biofilm to planktonic. The latter could be explained with the presence in EOs of small molecules presumably acting as quorum sensing inhibitors [28]. In any case, moderation on conclusion has to be undertaken since interactive and synergistic effects among the EO chemical comnponents—including minor ones—can affect biological potency. Application of a in house python based machine learning protocol led to definition of a classification model able to discriminate essential oils in active and inactive at a cut-off value of 50% of biofilm formation using a concentration of 48.8 µg/mL. Investigation of the most important components by means of feature importance and partial dependence plots seems to indicate estragole and phellandral as the chemical components mostly related to biofilm inhibition, while d-limonene, pulegone, and chrysanthenone seem to be related to biofilm production. The classification model as validated by five performance metrics are an example showing machine learning as tool to investigate complex chemical mixtures and possibly in prospective experiments it could enable scientists to understand the mechanism by which EOs act.

On the basis of this founding further experiments are on due course to investigate EOs rich in the above five chemical components to validate the classification model. As the main goal of this report was focused on the evaluation of antimicrobial and antibiofilm potencies of and an extensive number of different essential oils, no investigation was undertaken on EO effects on mature biofilms and their eradication.

The data from this study, enriched by further experiments carried out with other EOs and bacterial species, could enable the identification of blends of EOs specifically designed to obtain products with strong anti-biofilm efficacy applicable in many fields: airborne decontamination, products for dermatological and respiratory tract infections, etc.

## Figures and Tables

**Table 1:** Effect of EOs at different concentrations (scalar concentrations starting from 25 mg/mL) on biofilm formation of *P. aeruginosa* PaO1. Data are reported as percentage of residual biofilm after the treatment in comparison with the untreated one. Each data point is composed of four independent experiments each performed at least in triplicate.

EO (mg/mL)	R3	R12	CJM3	CAM4	CSM2	FS1	FSM5	FOM4
25	55.11	50.62	36.71	59.48	28.23	30.84	47.38	28.31
12.5	41.41	45.18	37.10	54.56	41.36	38.83	49.12	25.48
6.25	37.77	57.44	34.64	55.82	37.79	30.16	49.51	25.01
3.125	42.25	57.42	40.09	71.80	40.48	38.40	54.16	25.62
1.55	48.49	65.06	38.80	69.33	44.34	44.93	90.16	30.44
0.78	47.81	64.60	50.05	67.19	51.65	38.67	78.74	37.15
0.39	49.49	61.97	54.87	72.45	42.97	84.39	76.10	39.34
0.18	57.39	66.48	53.90	69.42	49.18	60.02	80.81	32.54
0.09	60.37	61.83	48.00	72.80	43.08	59.75	78.51	38.32
0.0488	70.65	59.05	52.99	83.24	50.26	42.44	88.71	38.28
0.0244	45.12	63.91	41.65	73.93	34.01	47.96	59.63	39.47
0.0122	64.81	66.11	46.59	73.19	40.02	57.26	75.63	38.29
0.0061	65.40	59.87	50.14	82.00	37.86	27.50	143.53	37.34
0.00305	63.06	78.37	45.22	69.05	35.44	38.70	117.45	39.75
0.00152	60.94	70.11	44.76	79.77	40.72	44.94	104.92	47.53
0.00076	61.95	65.18	40.29	73.17	37.14	37.13	112.35	53.00
0.0003814	61.13	62.05	49.74	76.76	47.15	42.07	113.75	37.23
0.0001907	56.98	65.80	48.48	83.21	49.49	36.59	90.67	57.15
0.00009535	72.29	65.27	45.52	71.44	52.18	39.25	79.33	46.41
0.000047675	64.71	74.79	44.19	91.78	46.23	43.30	99.52	68.74

**Table 2:** Cross-validation scores for the binary GB classification models ^a^.

Statistical Parameter	At 48.8 µg/mL	At 3.125 mg/mL
**ACC CV**	0.90	0.72
**MCC CV**	0.64	0.51
**Precision–Recall AUC**	0.84	0.72
**ROC AUC**	0.80	0.68

^a^ final optimized models were obtained with the following settings: max depth = 3, max features = 0.9, min samples_leaf = 16, *n* estimators = 500.

## Supplementary Materials

The following are available online.
