# Supplementary material for: Antimicrobial and Antibiofilm Activity and Machine Learning Classification Analysis of Essential Oils from Different Mediterranean Plants against Pseudomonas aeruginosa
Source: Molecules. 2018 Feb 23;23(2):482. doi: 10.3390/molecules23020482 (PMC6017904; doi:10.3390/molecules23020482)
Supplement: Supplementary file 1 [file molecules-23-00482-s001.pdf]

**Supplementary Material**

---

# Antimicrobial and antibiofilm activity and machine learning classification analysis of essential oils from different Mediterranean plants against *Pseudomonas aeruginosa*

Marco Artini[1,ç], Alexandros Patsilinakos[2,3,5,ç], Rosanna Papa[1], Mijat Božović[2,4], Manuela Sabatino[2,3], Stefania Garzoli[2], Gianluca Vrenna[1], Marco Tilotta[1], Federico Pepi[2], Rino Ragno[2,3,5,*]and Laura Selan[1]

[1] Department of Public Health and Infectious Diseases, Sapienza University, P.le Aldo Moro 5, 00185 Rome, Italy

[2] Department of Drug Chemistry and Technology, Sapienza University, P.le Aldo Moro 5, 00185 Rome, Italy; stefania.garzoli@uniroma1.it (S.G.), federico.pepi@uniroma1.it (F.P.)

[3] Rome Center for Molecular Design, Department of Drug Chemistry and Technology, Sapienza University, P.le Aldo Moro 5, 00185 Rome, Italy; mijat.bozovic@uniroma1.it (M.B.)

[4] Faculty of Natural Sciences and Mathematics, University of Montenegro, Džordža Vašingtona bb, 81000 Podgorica, Montenegro;

[5] Alchemical Dynamics s.r.l., 00125 Rome, Italy;

* Corresponding authors:
rino.ragno@uniroma1.it (R.R.), tel.: +39-6-4991-3937; fax: +39-6-4991-3627

[ç] M.A. and A.P. contributed equally to the paper

**Table S1**. GB parameters used in the grid search for optimal hyperparameterization

| | | | | | | | | |
|---|---|---|---|---|---|---|---|---|
| **N estimators** | 100 | 250 | 500 | 750 | 1000 | 1250 | 1500 | 1750 |
| **Max depth** | 3 | 4 | 5 | 6 | 7 | 8 | 9 | 10 |
| **Min samples leaf** | 1 | 3 | 5 | 7 | 9 | 11 | 13 | 15 |
| **Max features** | 0.7 | 0.6 | 0.5 | 0.4 | 0.3 | 0.2 | log2[a] | sqrt[b] |

[a] sqrt: max features = sqrt(n features). [b] log2: max features = log2(n features)

**Table S2.** Relative yields % of EOs over time.

| Plant species | **h** [1] | **1** | **2** | **3** | **6** | **12** | **24** |
|---|---|---|---|---|---|---|---|
| | ***m*** [2] | | | | | | |
| **CG** | *Jul.* | 0.300 | 0.350 | 0.360 | 0.366 | 0.370 | 0.373 |
| | *Aug.* | 0.300 | 0.360 | 0.400 | 0.420 | 0.426 | 0.432 |
| | *Sep.* | 0.190 | 0.250 | 0.300 | 0.360 | 0.376 | 0.381 |
| | *Oct.* | 0.180 | 0.260 | 0.290 | 0.320 | 0.328 | 0.328 |
| **FV** | *Aug.* | 0.070 | 0.110 | 0.140 | 0.180 | 0.196 | 0.213 |
| | *Sep.* | 0.090 | 0.140 | 0.170 | 0.200 | 0.218 | 0.240 |
| | *Oct.* | 0.360 | 0.640 | 0.830 | 1.090 | 1.210 | 1.250 |
| **RS** | *na* | 0.200 | 0.300 | 0.440 | 0.640 | 0.740 | 0.800 |

[1] Extraction hour, [2] Month of harvesting, na – not applicable.

**Table S3.** EO Yield % calculated on the dried (FV and RS) or fresh (CG) plant material.

| Plant species | h [1] / *m* [2] | 1 | 2 | 3 | 6 | 12 | 24 |
|---|---|---|---|---|---|---|---|
| **CG** | *Jul.* | 0.300 | 0.050 | 0.010 | 0.006 | 0.004 | 0.003 |
| | *Aug.* | 0.300 | 0.060 | 0.040 | 0.020 | 0.006 | 0.006 |
| | *Sep.* | 0.190 | 0.060 | 0.050 | 0.060 | 0.016 | 0.005 |
| | *Oct.* | 0.180 | 0.080 | 0.030 | 0.030 | 0.008 | 0.0004 |
| **FV** | *Aug.* | 0.070 | 0.040 | 0.030 | 0.040 | 0.016 | 0.017 |
| | *Sep.* | 0.090 | 0.050 | 0.030 | 0.030 | 0.018 | 0.022 |
| | *Oct.* | 0.360 | 0.280 | 0.190 | 0.260 | 0.120 | 0.040 |
| **RS** | *na* | 0.200 | 0.100 | 0.140 | 0.200 | 0.100 | 0.060 |

[1] Extraction hour, [2] Month of harvesting, na – not applicable.

**Table S4.** Chemical composition (%) of the most active FVEO samples.

| # [1] | Name | Sample [2] | | | | | | | | | | |
|---|---|---|---|---|---|---|---|---|---|---|---|---|
| | | **A1h** | **A3h** | **A6h** | **A12h** | **AM1** | **AM2** | **AM3** | **AM 4** | **S1h** | **OM1** | **OM4** |
| **1** | *α*-pinene | - | 2.9 | 4.9 | 1.1 | 1.0 | 7.0 | 3.9 | 3.3 | 1.9 | 4.3 | 19.6 |
| **2** | *β*-pinene | - | - | - | - | - | - | - | - | - | 3.3 | 1.4 |
| **3** | *β*-terpinene | - | - | - | - | - | 0.4 | 0.9 | 0.6 | - | - | - |
| **4** | *β*-myrcene | 1.6 | 1.4 | 1.5 | 0.8 | 0.2 | 0.4 | 1.0 | 1.1 | 1.4 | 3.0 | 2.0 |
| **5** | *α*-phellandrene | 18.3 | 6.1 | 20.5 | 3.9 | 0.4 | 0.7 | 1.0 | 10.5 | 13.0 | 2.7 | 7.7 |
| **6** | d-limonene | 7.5 | 7.0 | 8.2 | 3.7 | 1.8 | 3.4 | 6.8 | 6.0 | 12.4 | 1.0 | 4.4 |
| **7** | *β*-phellandrene | 6.6 | 4.1 | 4.6 | 2.2 | 1.2 | 2.2 | 5.0 | 4.4 | 6.8 | 1.1 | 1.6 |
| **8** | *γ*-terpinene | 1.1 | 1.1 | 2.6 | 0.8 | 0.1 | 0.2 | 1.0 | 1.1 | 1.5 | 3.9 | 1.6 |
| **9** | *o*-cymene | 35.5 | 25.2 | 19.1 | 14.0 | 16.0 | 23.5 | 35.3 | 28.7 | 52.5 | 1.5 | 2.9 |
| **10** | terpinolene | - | - | - | - | - | - | - | - | - | 0.1 | 0.5 |
| **11** | fenchone | 2.4 | 1.3 | 0.4 | 0.2 | 4.8 | 2.6 | 2.0 | 1.7 | - | 4.8 | 8.8 |
| **12** | dehydro-p-cymene | 0.2 | 0.5 | 0.6 | 1.8 | - | - | - | - | - | - | - |
| **13** | isomenthone | 0.5 | 1.1 | 1.0 | 2.2 | - | - | - | - | - | - | - |
| **15** | pulegone | 2.3 | 4.4 | 0.3 | 9.3 | 4.5 | 2.2 | 2.8 | 3.7 | 0.4 | - | - |
| **16** | estragole | 18.5 | 14.5 | 6.1 | 1.7 | 22.5 | 14.8 | 14.6 | 13.2 | - | 70.6 | 47.1 |
| **17** | *p*-ment-en-2-one | - | 3.4 | 3.5 | 9.7 | 3.8 | 1.1 | 2.0 | 2.7 | 0.6 | - | - |
| **18** | phellandral | - | 1.1 | 2.3 | 4.1 | 1.4 | 1.6 | 1.1 | 1.3 | 0.2 | - | - |
| **20** | *cis*-sabinol | 4.7 | 8.6 | 6.3 | 9.5 | 7.8 | 5.6 | 6.1 | 6.5 | 2.2 | - | - |
| **21** | *p*-cymen-8-ol | - | 3.0 | 1.7 | 3.5 | 5.2 | 2.0 | 1.8 | 1.8 | 0.8 | - | 1.1 |
| **22** | 2,3-pinanediol | - | - | - | - | 7.8 | 3.3 | 2.8 | 5.7 | 1.3 | - | - |
| **26** | thymol | - | 5.9 | 9.1 | 14.7 | 4.8 | 6.7 | 3.7 | 4.6 | 1.2 | - | - |
| **27** | myristicin | - | - | 2.2 | 5.6 | 1.6 | 0.8 | 1.0 | - | 2.7 | 1.2 | 1.1 |
| **28** | piperitenone oxide | - | - | 2.5 | 5.8 | 4.9 | 19.5 | 4.0 | - | - | 0.7 | - |
| | Unidentified compounds | **0.8** | **8.4** | **2.6** | **5.4** | **10.2** | **2.0** | **3.2** | **3.1** | **1.1** | **1.8** | **0.2** |

[1] # indicates the compound identification number; [2] samples names were given by merging the month first letter and extraction time as reported in Table S2 or by merging the first letter of the month, the letter M (mixture) and serial number of the mix.

**Table S5.** Chemical composition (%) of the most active RSEO samples.

| # [1] | Name | Sample [2] | | | |
|---|---|---|---|---|---|
| | | **1h** | **3h** | **12h** | **30h** |
| **1** | *α*-pinene | 3.9 | 3.6 | 1.2 | - |
| **2** | *β*-pinene | 4.6 | 3.4 | 1.5 | 0.1 |
| **3** | *β*-myrcene | 0.9 | 1.2 | 0.4 | - |
| **4** | *α*-phellandrene | - | - | 5.4 | 0.6 |
| **5** | *d*-limonene | 7.4 | 6.7 | 1.2 | 0.3 |
| **6** | *β*-terpinene | 3.0 | 4.9 | 1.8 | 0.3 |
| **7** | *β*-ocimene | 0.5 | 1.3 | 0.8 | 0.2 |
| **8** | *o*-cymene | 40.1 | 3.8 | 7.4 | 4.2 |
| **9** | terpinolene | - | 2.1 | 1.6 | - |
| **11** | borneol | - | 3.3 | 3.0 | 3.0 |
| **12** | pulegone | - | - | 0.8 | 2.6 |
| **13** | citral | - | 1.0 | - | - |
| **14** | cryptone | - | 2.5 | - | - |
| **15** | *p*-menth-1-en-2-one | - | 2.2 | 2.1 | 9.7 |
| **17** | *cis*-sabinol | - | 5.8 | 4.3 | 12.9 |
| **18** | *p*-cymen-8-ol | 9.2 | 13.4 | 3.0 | 6.4 |
| **19** | piperitenone oxide | 6.5 | 3.6 | 1.0 | 1.9 |
| **21** | 2,3-pinanediol | 9.6 | 1.8 | 2.1 | - |
| **23** | myristicin | - | - | 3.2 | 1.7 |
| **24** | apiol | 6.5 | 21.1 | 59.2 | 56,1 |
| | Unidentified compounds | **7.8** | **18.3** | **0.0** | **0.0** |

[1] # indicates the compound identification number; [2] samples names indicate the extraction time as reported in Table S1.

**Table S6**. Arbitrary classification of 89 EOs samples in 4 different classes depending on their capability to impair biofilm formation.

| **Strong reduction (< 40% residual biofilm)** | | | **Medium reduction (40-80% residual biofilm)** | | | **No reduction (80 - 100% residual biofilm)** | | | **Enhancer effect (>100% residual biofilm)** | | |
|---|---|---|---|---|---|---|---|---|---|---|---|
| **FV** | **CG** | **RS** | **FV** | **CG** | **RS** | **FV** | **CG** | **RS** | **FV** | **CG** | **RS** |
| FA1 | COM2 | | FA2 | CJM3 | R1 | FO1 | CA6 | RM1 | | CA1 | |
| FA3 | | | FA24 | CJM4 | R2 | | CJ3 | RM3 | | CA2 | |
| FA6 | | | FAM5 | CO1 | R3 | | CJM1 | RM4 | | CA3 | |
| FA12 | | | FS2 | CO2 | R6 | | CJM5 | | | CAM1 | |
| FAM1 | | | FS3 | CO3 | R12 | | COM1 | | | CAM3 | |
| FAM2 | | | FS6 | CO6 | R24 | | COM3 | | | CJ1 | |
| FAM3 | | | FS12 | CO12 | R30 | | COM5 | | | CJ2 | |
| FAM4 | | | FS24 | CO24 | RM2 | | CS2 | | | CJM2 | |
| FS1 | | | FSM2 | CS1 | RM5 | | CS3 | | | COM4 | |
| FOM1 | | | FSM3 | CS12 | RM6 | | CSM1 | | | CS6 | |
| FOM4 | | | FSM4 | CS24 | | | | | | CSM3 | |
| | | | FSM5 | CSM2 | | | | | | CSM5 | |
| | | | FO2 | CSM4 | | | | | | | |
| | | | FO3 | CA12 | | | | | | | |
| | | | FO6 | CA24 | | | | | | | |
| | | | FO12 | CAM2 | | | | | | | |
| | | | FO24 | CAM4 | | | | | | | |
| | | | FOM2 | CAM5 | | | | | | | |
| | | | FOM3 | CJ6 | | | | | | | |
| | | | FOM5 | CJ12 | | | | | | | |
| | | | | CJ24 | | | | | | | |

FV: *Foeniculum vulgare*; CG: *Calamintha nepeta* subsp. *glandulosa*; RS: *Ridolfia segetum*.

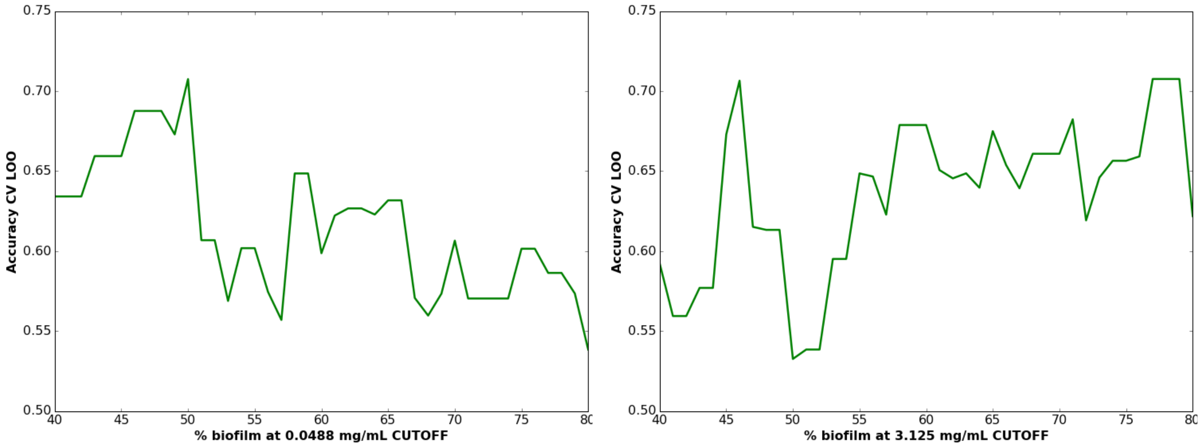


**A** **B**

**Figure S1**. Analysis of best cutoff values for the GB classification models. A: at 48.8 μg/ml; B: at 3.125 mg/ml.

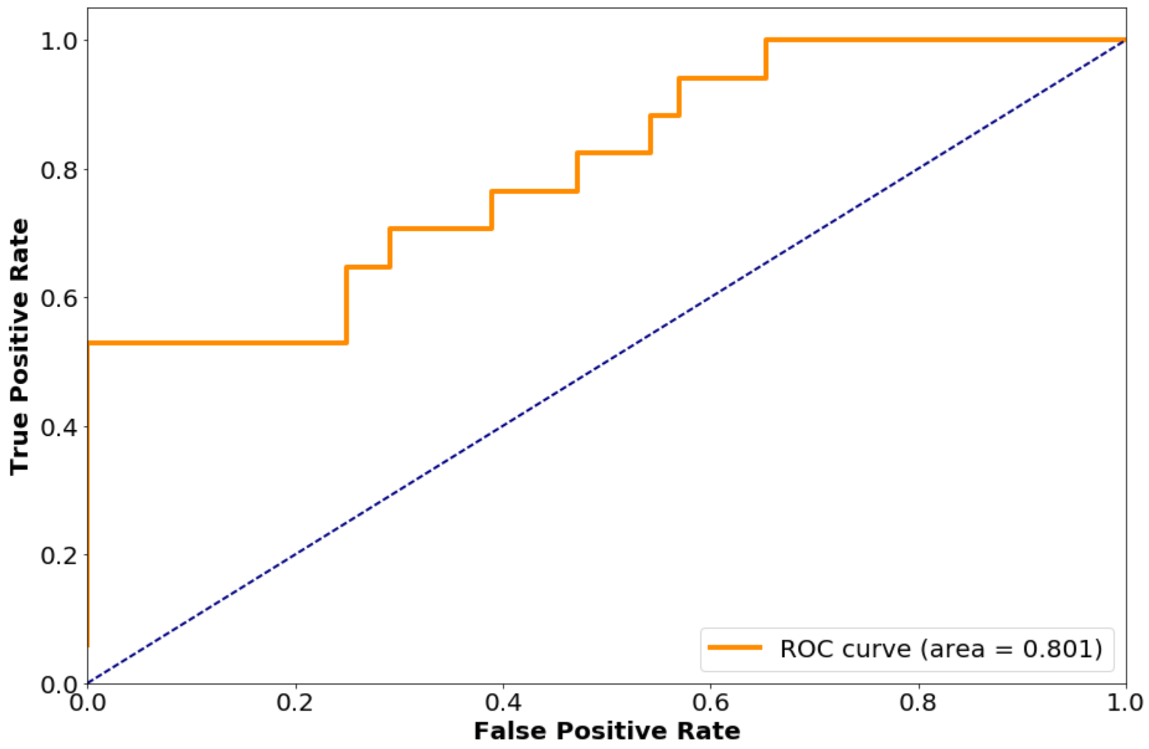


**Figure S2**. ROC curve for the GB classification model obtained for biofilm inhibition measured at 48.8 μg/ml.

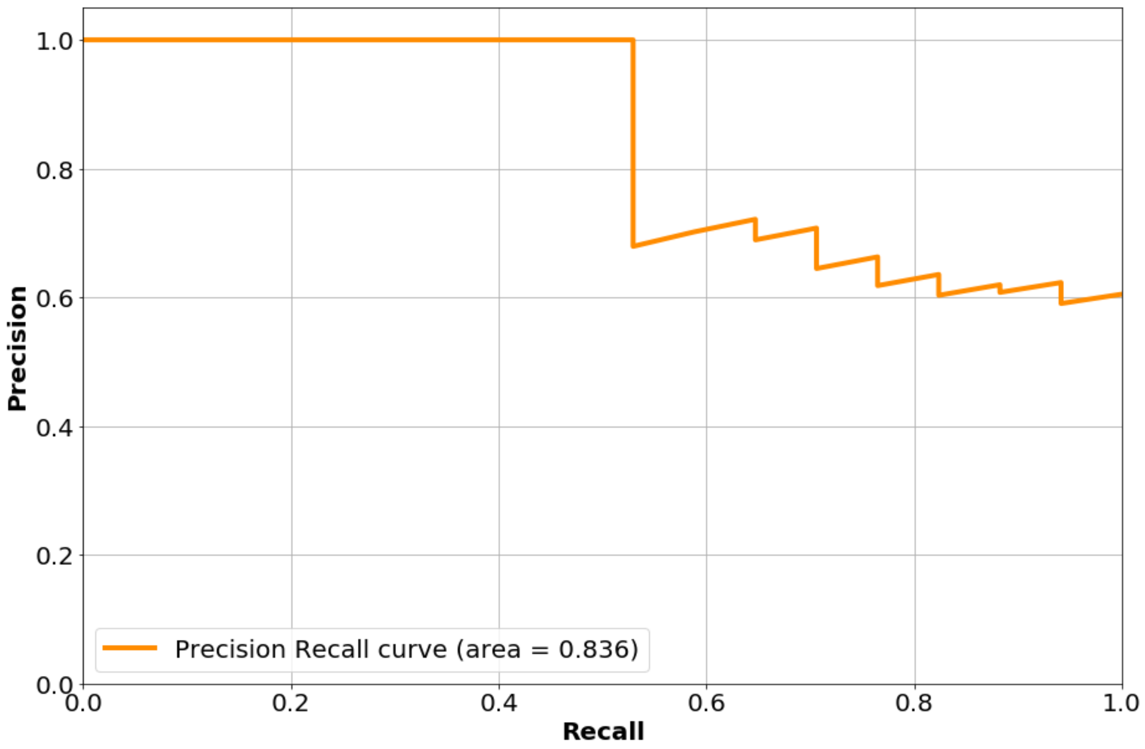


**Figure S3**. Precision Recall curve for the GB classification model obtained for biofilm inhibition measured at 48.8 μg/ml.

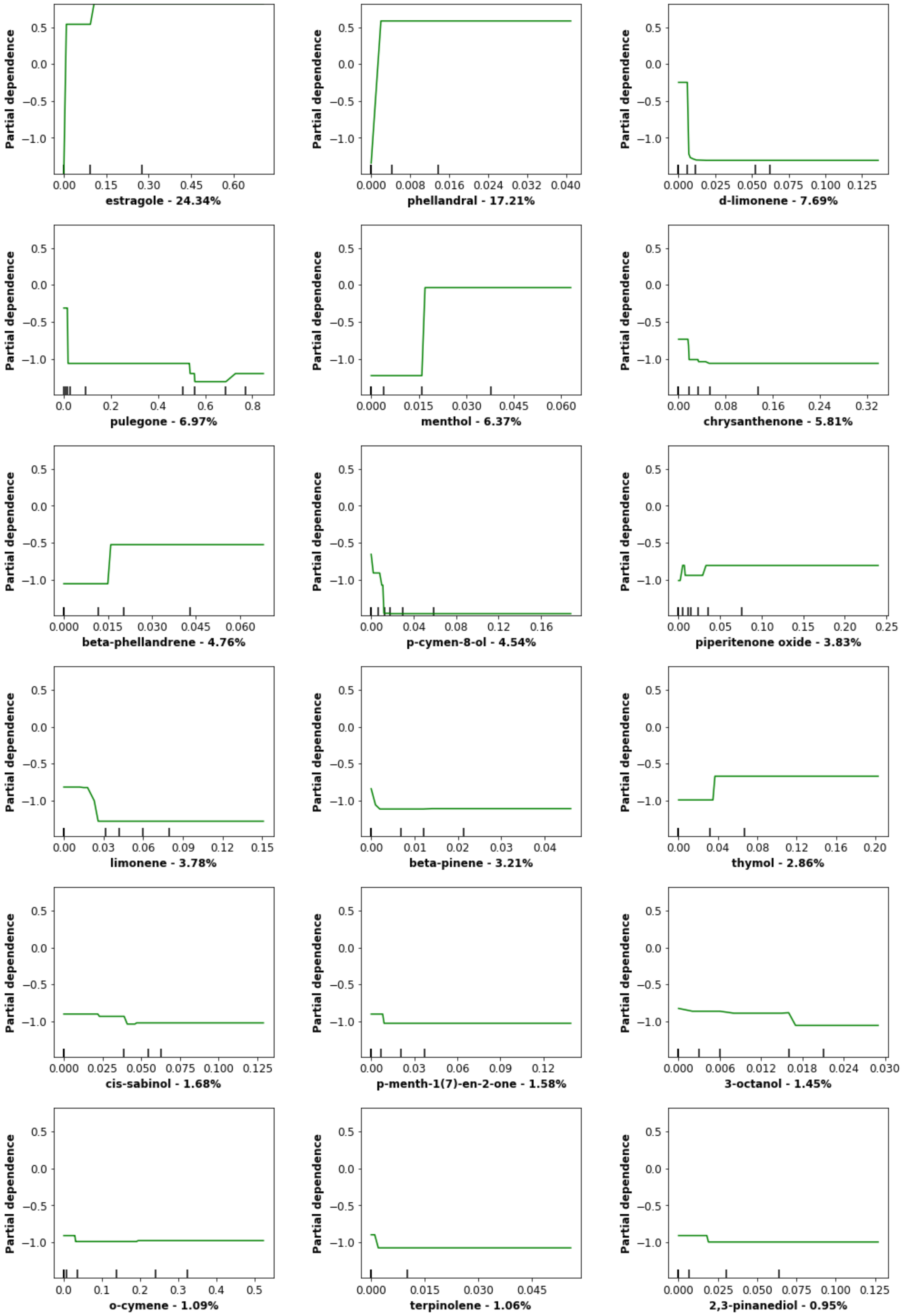


**Figure S4.** Partial Dependence plot obtained for the GB classification models at 48.8 μg/ml for the first 18 EOs' chemical components

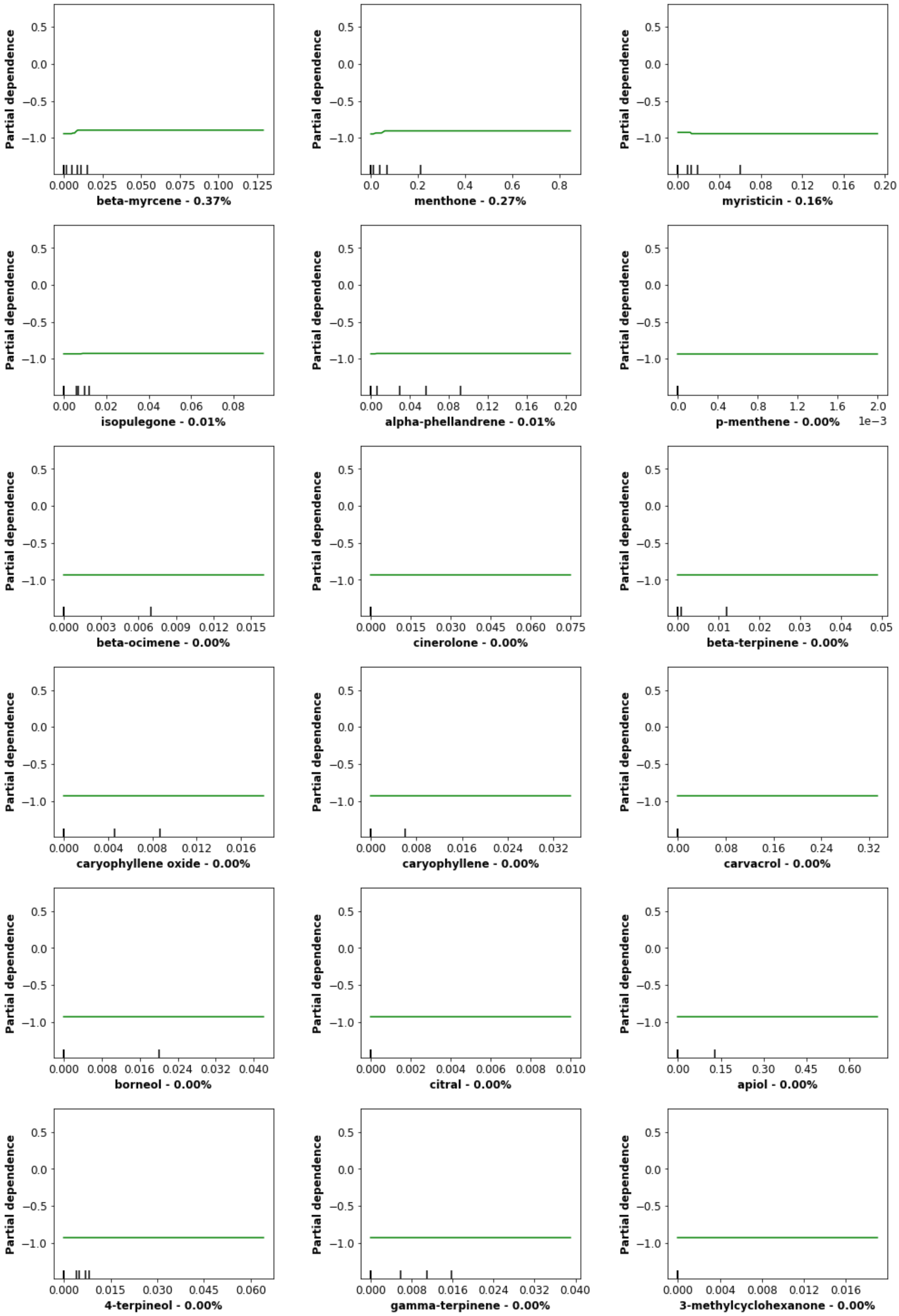


***continue*** **Figure S4.** Partial Dependence plot obtained for the GB classification models at 48.8 µg/ml for the second 18 EOs' chemical components

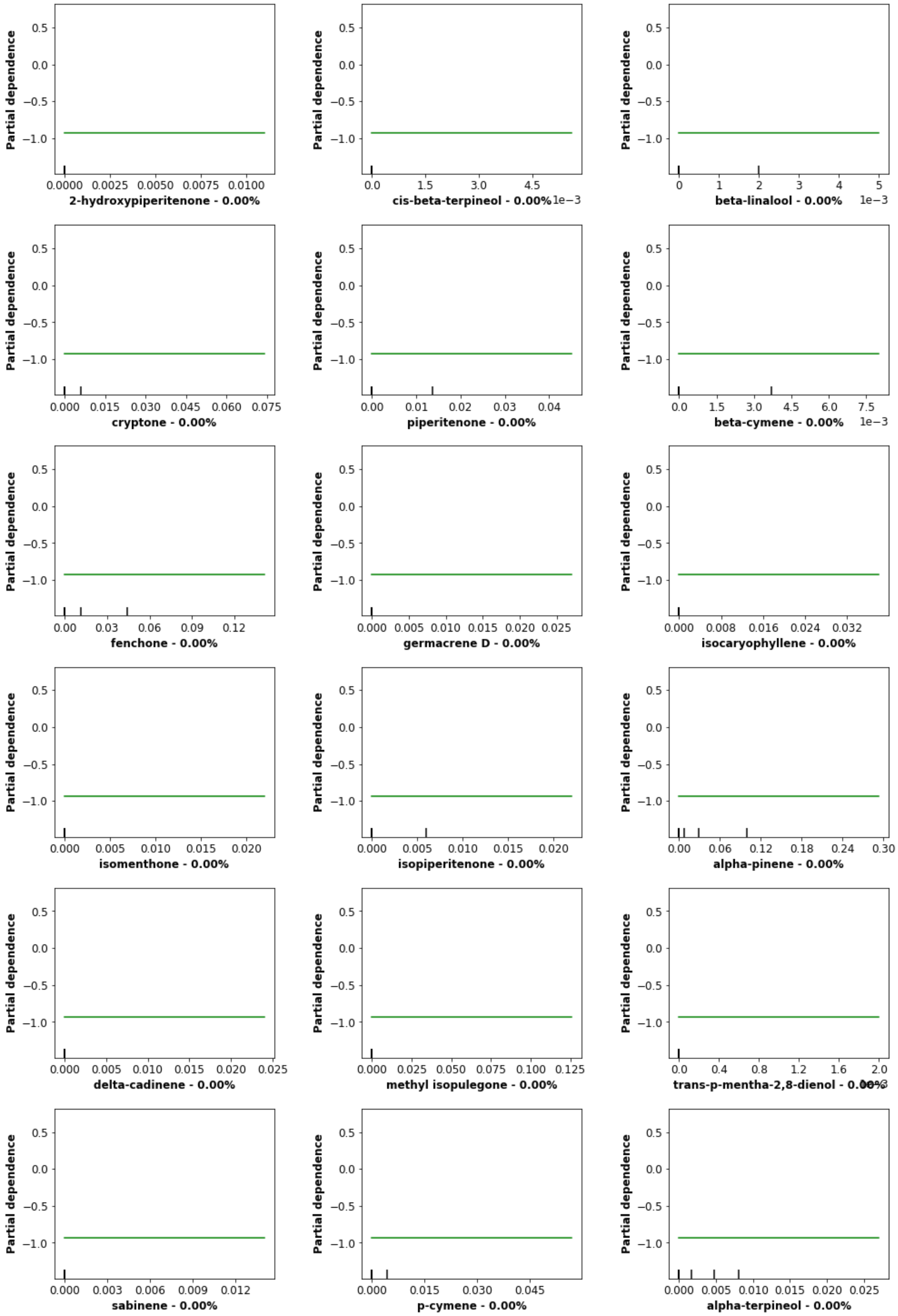


***continue*** **Figure S4.** Partial Dependence plot obtained for the GB classification models at 48.8 μg/ml for the last 18 EOs' chemical components

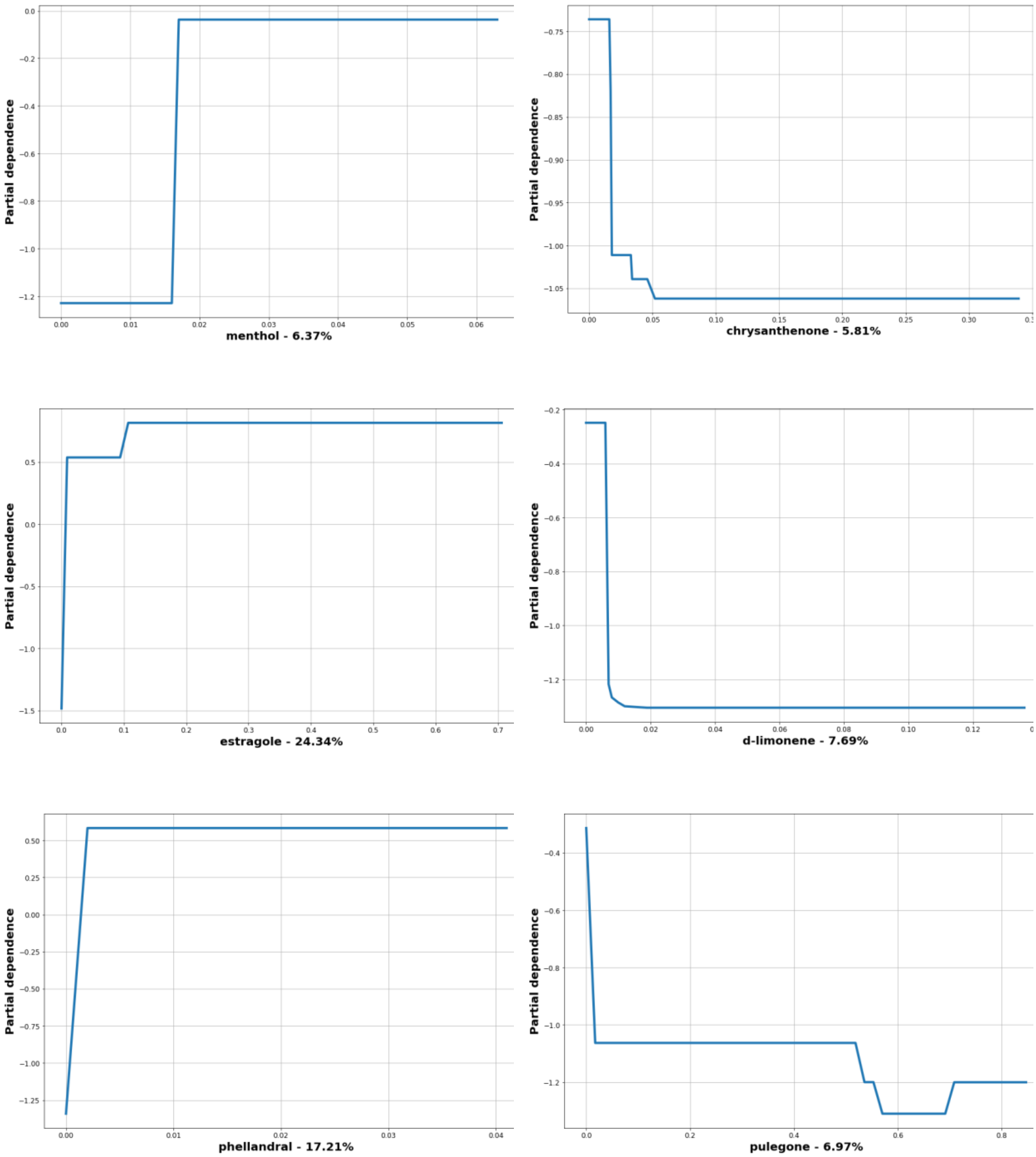


**Figure S5**. Partial Dependence plot obtained for the GB classification models at 48.8 μg/ml for the most important chemical components.
